# Cytologic and Molecular Diagnostics for Vitreoretinal Lymphoma: Current Approaches and Emerging Single-Cell Analyses

**DOI:** 10.3389/fmolb.2020.611017

**Published:** 2021-01-11

**Authors:** Wei Jian Tan, Mona Meng Wang, Paola Ricciardi-Castagnoli, Anita Sook Yee Chan, Tong Seng Lim

**Affiliations:** ^1^A. Menarini Biomarkers Singapore Pte. Ltd., Singapore, Singapore; ^2^Translational Ophthalmic Pathology Platform, Singapore Eye Research Institute, Singapore, Singapore; ^3^Singapore National Eye Centre, Singapore, Singapore

**Keywords:** vitreoretinal lymphoma, diffuse large B-cell lymphoma, cytology, molecular diagnostics, single-cell analysis, precision medicine

## Abstract

Vitreoretinal lymphoma (VRL) is a rare ocular malignancy that manifests as diffuse large B-cell lymphoma. Early and accurate diagnosis is essential to prevent mistreatment and to reduce the high morbidity and mortality associated with VRL. The disease can be diagnosed using various methods, including cytology, immunohistochemistry, cytokine analysis, flow cytometry, and molecular analysis of bulk vitreous aspirates. Despite these options, VRL diagnosis remains challenging, as samples are often confounded by low cellularity, the presence of debris and non-target immunoreactive cells, and poor cytological preservation. As such, VRL diagnostic accuracy is limited by both false-positive and false-negative outcomes. Missed or inappropriate diagnosis may cause delays in treatment, which can have life-threatening consequences for patients with VRL. In this review, we summarize current knowledge and the diagnostic modalities used for VRL diagnosis. We also highlight several emerging molecular techniques, including high-resolution single cell-based analyses, which may enable more comprehensive and precise VRL diagnoses.

## Introduction

Primary vitreoretinal lymphoma (PVRL), previously known as primary intraocular lymphoma or ocular reticulum cell sarcoma, was originally described by Cooper and Riker (1951) in 1951. This rare disorder usually involves the retina, the vitreous, or both structures. Blurred vision and ataxia are the most common symptoms that prompt individuals with PVRL to seek ophthalmic examination. PVRL is considered a manifestation of primary central nervous system lymphoma (PCNSL); however, only 15–28% of PCNSL cases have intraocular involvement, while 65–90% of PVRL cases subsequently develop a CNSL (Chan et al., 2011; Hong et al., 2011). VRL has also been identified in patients with systemic lymphoma (Salomao et al., 2013). Therefore, in this review, we focused on both primary and secondary manifestations of VRL.

Most (>90%) VRL cases are of B-cell origin, but rare cases of T-cell and natural killer (NK) cell VRL have been reported (Coupland et al., 2005a; Maruyama et al., 2015; Fend et al., 2016). Therefore, this fatal, high-grade non-Hodgkin lymphoma is pathologically classified as a diffuse large B-cell lymphoma (DLBCL) by the World Health Organization (Coupland et al., 2004). In a landmark publication in 2000, Alizadeh et al. (2000) used gene expression profiling to identify three DLBCL molecular subtypes, namely, activated B-like DLBCL (ABC), germinal center B-cell (GCB), and primary mediastinal DLBCL. Based on this stratification according to molecular signatures, PCNSL was subsequently classified as ABC DLBCL (Camilleri-Broet et al., 2006; Bhagavathi et al., 2008; Cheng et al., 2008; Montesinos-Rongen et al., 2009; Hattab et al., 2010; Li et al., 2017; Belhouachi et al., 2020). Indeed, >80% of VRL cases belong to this subtype (Araujo and Coupland, 2017; Karakawa et al., 2018; Chen et al., 2019; Fan et al., 2019).

According to a report by the United States National Cancer Institute, the incidence of PCNSL more than doubled in a decade, from 2.7 cases per ten million people in 1973–1974 to 7.5 cases per 10 million people in 1982–1984 (Eby et al., 1988); numbers then further surged to 100 cases per 10 million people by the early nineties (Schabet, 1999). This increase in incidence affected both immunocompetent and immunocompromised patients (Tuaillon and Chan, 2001), as confirmed by a Statistical Report produced by the Central Brain Tumor Registry of the US in the early 2000's, which estimated that immunocompetent patients alone accounted for 46 cases per ten million people (Levasseur et al., 2013). As of 2017, the incidence of PCNSL has increased four times since the initial findings reported in 1973. Most worryingly, the overall survival of patients with PCNSL has not changed in four decades, despite general improvements in healthcare systems worldwide (Mendez et al., 2018).

As a subset of PCNSL, VRL cases remain rare but have nonetheless seen a similar increasing trend over the past four decades, particularly in Western countries (Chan et al., 2002; Levy-Clarke et al., 2005; Levasseur et al., 2013; Sagoo et al., 2014). An extensive retrospective study involving 16 centers in seven countries found that VRL can affect individuals at almost any age in adulthood (ranging from 24 to 85 years), but the median age of diagnosis was 63 years in immunocompetent patients (Grimm et al., 2007). However, VRL can also affect pediatric patients aged <16 years (Wender et al., 1994; Chan et al., 2000; Sobrin et al., 2005). There are conflicting data with regard to VRL sex differences: some studies have reported a higher prevalence in women (Buettner and Bolling, 1993; Kempin et al., 2018), whereas others have reported a higher prevalence in men (Chan and Wallace, 2004; Fend et al., 2016).

Given the increasing prevalence of PCNSL and VRL, which has not been matched with improvements in patient outcomes, more medical attention and further clinical studies of these diseases are clearly warranted. Over the past two decades, there has been considerable interest in the application of cytological and molecular assays to improve the accuracy of diagnosing VRL. In this article, we review the current application of these assays. We will also discuss future opportunities for emerging technologies, including single cell-based analyses that enable a comprehensive and precise VRL diagnosis.

## Vitreoretinal Lymphoma (VRL) Diagnostic Approaches

The clinical manifestations of VRL include blurred vision, decreased visual acuity, red eye, and the appearance of floaters (Hoffman et al., 2003; Chan et al., 2011). These symptoms are often subtle and not specific to VRL, given that they can also be found in other eye diseases (Gariano and Kim, 2004; Guerriero et al., 2011; van Laar et al., 2015). While fundus examination and imaging are not definitive and not used as a confirmatory diagnostic tool, they can serve as a preliminary examination in a suspected VRL case. As malignant cells populate the vitreous cavity, VRL presents as diffuse infiltrates clouding the fundus upon fundoscopic examination (Davis, 2013). Imaging techniques, such as autofluorescence, fluorescein angiography, optic coherence tomography, and B-scan ultrasound, can also be used as differential testing approaches to identify VRL clinical features (Wang et al., 2011). Two clinical characteristics of VRL are worth noting: (1) “aurora borealis,” when malignant cells line up along the vitreal fibers; and (2) “leopard spot,” when malignant cells gather and form subretinal pigmented lesions (Chan et al., 2011).

Despite the utility of the abovementioned physical modalities for differential diagnostic purposes, VRL is a uveitis masquerade syndrome, i.e., it mimics the clinical features of uveitis, which could potentially lead to a misdiagnosis (Corriveau et al., 1986; Rothova et al., 2001; AlQahtani et al., 2014). As such, a definitive VRL diagnosis requires the direct visualization of malignant cells *via* cytology—the current gold standard for VRL diagnosis (Fend et al., 2016). Below, we outline various VRL diagnostic techniques, including cytology (and immunocytochemistry), cytokine analysis, flow cytometry, and molecular analysis.

### Cytology and Immunocytochemistry-Based Diagnostics

Proper anatomical sampling is critical for an accurate, cytology-based diagnosis of VRL. In this regard, sampling the vitreous fluid offers the highest chance of collecting lymphoma cells that reside in the retina and subretinal space and is preferred over the anatomically distanced aqueous humor. Vitreous fluid sampling is typically conducted by either vitreous aspiration or vitrectomy (Venkatesh et al., 2019), both techniques that are technically challenging. Specifically, the low cellularity of the vitreous fluid means that repeated sampling may be required, thereby causing distress to the patient as well as conferring a risk for ocular complications (Fend et al., 2016).

Not surprisingly, cytological examination requires specialist training and the experience of a pathologist, and accurate interpretation depends on the quality and integrity of vitreous biopsies. Hematoxylin and eosin, Giemsa, and Diff-Quik stains are commonly used (Coupland and Damato, 2008) to help identify malignant cells; with VRL, malignant cells exhibit large, pleomorphic nuclei, scant basophilic cytoplasm staining, and are two- to four times larger than normal lymphocytes (Chan, 2003; Coupland et al., 2004; Farkas et al., 2004). The presence of contaminants, debris, necrotic cells, and infiltrating immunoreactive cells, as well as the use of corticosteroids, can complicate the interpretation of staining, resulting in a high rate (70%) of false-negative diagnoses (Char et al., 1988b; Whitcup et al., 1993; Coupland et al., 2003). Davis et al. (2005) found that such confounders also resulted in the poor positive predictive value of cytology (30.8%), with malignant cells being detected in just 4/13 confirmed VRL cases.

Malignant cells are particularly fragile and are thus challenging to isolate and culture for downstream analyses. Careful sample handling is thus essential to ensure accurate VRL diagnosis. As malignant cells have limited *ex vivo* viability, they begin to undergo apoptosis and morphological degradation within just 60 min of vitreous aspiration (Char et al., 1988a; Gonzales and Chan, 2007; Chan et al., 2011; Mehta et al., 2015; Ranty et al., 2015). Indeed, a study by Wang et al. (2011) found no identifiable cells in 23/182 (12%) of collected samples, preventing the use of cytologic analyses to make a VRL diagnosis in these cases. Therefore, ocular specimens should be handled rapidly and appropriately; if prompt diagnosis is not feasible, samples should be placed in an appropriate fixative to preserve cytological details (Coupland, 2012; Davis, 2013; Wang et al., 2019). Nonetheless, cytology when used as a stand-alone test can confirm VRL in 45%−60% of cases, with a low occurrence of false positives (Davis et al., 2005; Wittenberg et al., 2008; Kimura et al., 2012).

The sensitivity of a cytologic diagnosis can be enhanced by complementing it with immunocytochemical staining. Here, an antibody is used to detect cell type-specific markers (Coupland et al., 2003), namely, B-cell markers (e.g., CD20, CD79a, and PAX5) and a T-cell marker (e.g., CD3). When combined, these markers can help delineate malignant cell types, while Ki-67 staining can be used to detect the unusually high (>80%) proliferation rate of malignant cells (Fend et al., 2016). Undiluted vitreous obtained *via* dry anterior vitrectomy is the optimal material for a conventional smear cytology examination. With recent advances in pathological techniques, cell block preparation using infusion fluids obtained during vitreous surgery could also be a useful complement for VRL diagnosis (Zaldivar et al., 2004). Recently, cytological examinations with high sensitivity and a low false-positive rate have been achieved when using diluted vitreous cell-block samples, highlighting the usefulness of cytology and the potential use of diluted biopsies to increase diagnostic yield (Kase et al., 2016).

### Cytokine Analyses

Interleukin-6 (IL-6) is a pro-inflammatory cytokine that is secreted under inflammatory conditions, including in the context of uveitis (Murray et al., 1990). By contrast, IL-10 is an anti-inflammatory cytokine that promotes B-cell lymphoma cell proliferation and inhibits apoptosis (Alas and Bonavida, 2001; Gupta et al., 2012). IL-10 also functions as an immunosuppressive cytokine, dampening immune responses to malignant cells in the eye (Davis, 2013). Currently, enzyme-linked immunosorbent assays (ELISAs) or cytokine multiplex assays are used to determine IL-10 and IL-6 levels in the vitreous and aqueous humor (Cassoux et al., 2007). This use of the IL-10:IL-6 ratio is based on the observation by Chan et al. (1995) of elevated IL-10 levels in the vitreous fluid of patients with VRL and a similar observation in patients with PCNSL by Whitcup et al. (1997). Following these two initial reports, various subsequent studies confirmed that ~90% of patients with VRL had an IL-10:IL-6 ratio >1 (Ohta et al., 2009; Wang et al., 2011; Kimura et al., 2012). A study by Cassoux et al. (2007) furthermore proposed an IL-10 cutoff value of >400 pg/ml (for vitreous) and >50 pg/ml (for aqueous humor) for suspected VRL cases.

IL-10 levels might also serve as a prognostic biomarker, as elevated IL-10 levels seem to be correlated with PCNSL and VRL aggression (Ramkumar et al., 2012) and shortened disease-free survival (Gupta et al., 2012). However, cytokine analyses cannot serve as a definitive diagnostic tool because, for example, cytokine levels can be affected by corticosteroid and immunosuppressant treatment. Furthermore, cases of VRL with an IL-10:IL-6 ratio <1 or even negative for IL-10 have been reported (Buggage et al., 1999; Akpek and Foster, 2000; Sugita et al., 2009; Wang et al., 2011). Moreover, Akpek et al. (1999) found that >60% of non-neoplastic uveitis cases had an IL-10:IL-6 ratio >1. Therefore, we do not support the use of cytokine profiling as the sole method for making a VRL diagnosis, but rather as an adjunctive tool to support VRL diagnosis (Chan et al., 1995; Cao et al., 2011).

### Flow Cytometry

Flow cytometry is a technique used to detect phenotype cells stained with fluorescent antibodies or dyes. The use of flow cytometry for VRL diagnosis was pioneered by Davis et al. (1997) in 1997, who found an improvement in VRL diagnosis from 30% based on cytology alone to 70% with the use of flow cytometry. A flow cytometry-based diagnosis of VRL is founded on the hypothesis that skewed expression of either immunoglobulin kappa (IGK) or lambda (IGL) light chains indicates the establishment of a restricted B-cell population and is suggestive of clonality in B-cell lymphoma (Horna et al., 2011). By definition, monotypic expression and immunoglobulin light-chain restriction is established when the IGK:IGL ratio is either >3 or <0.6 (Davis et al., 1997; Davis, 2013). The advantage of flow cytometry is its ability to simultaneously immunophenotype both B- and T-cell subsets, such as CD4^+^ and CD8^+^ T cells (Davis et al., 2012). Indeed, this approach has a reported sensitivity of 82% and specificity of 100%, which is comparable in terms of sensitivity to IL-10:IL-6 cytokine and immunoglobulin heavy chain (IGH) rearrangement analyses (Missotten et al., 2013).

Despite the favorable diagnostic sensitivity and specificity of flow cytometry, the use of this technique alone might not be sufficient as some cases of VRL are negative for clonal populations (Zaldivar et al., 2004). Moreover, surface immunoglobulin light chains can be lost in DLBCL, limiting the usefulness of the IGK:IGL ratio for clonality assessments (de Martini et al., 1988; Kaleem et al., 2000; Tomita et al., 2009). Similar to other diagnostic techniques, flow cytometry has a limited capacity to discriminate VRL from uveitis when reactive B- and T-cell infiltrates are present (Davis et al., 2012). Given that flow cytometry requires a large number of cells (relative to cytology), priority must be given to cytological examination for paucicellular ocular biopsies. This point alone hinders the widespread application of flow cytometry for VRL diagnosis (Sharara et al., 2003; Wang et al., 2011). Going forward, a combined approach, involving rare cell isolation by flow cytometry followed by downstream molecular analyses, will be necessary to improve the accuracy of VRL diagnosis.

### Molecular Testing

Molecular testing for VRL greatly accelerated following the development of the polymerase chain reaction (PCR) (Mullis et al., 1986). For VRL diagnosis, PCR, along with its derivatives such as AS-PCR (allelic-specific PCR) and droplet digital PCR (ddPCR), is used either independently or in combination with Sanger sequencing and high-resolution melt analyses. In contrast to cytology, molecular testing does not require expert interpretation and is thus considered less subjective. Other advantages of molecular testing include the ease of handling samples, rapid turnaround time, and the relatively low number of cells required compared with the numbers needed for cytology and flow cytometry. Furthermore, even archived samples, such as formalin-fixed paraffin-embedded (FFPE) tissue blocks, where the DNA quality therein is usually fragmented and of low quality (van Dongen et al., 2003; Kase et al., 2016), can be assessed using molecular testing methods. To date, most molecular testing has been conducted on bulk samples, focusing on disease biomarkers such as B- and T-cell receptor clonality, B-cell lymphoma 2 (BCL2), and myeloid differentiation primary response 88 (MYD88). Due to the inherent cellular heterogeneity of bulk samples, we anticipate there will be more emphasis on interrogating these disease biomarkers at single-cell resolution in the near future.

#### B-Cell and T-Cell Receptor Clonality

B-cell receptor clonality [pioneered by Katai et al. (1997)] was the first molecular biomarker established for VRL diagnostics. The premise of B-cell receptor clonality analysis is based on the fact that B cells function as antigen-presenting cells and confer humoral immunity *via* antibody secretion (Parkin and Cohen, 2001; Pieper et al., 2013). To ensure the recognition of a specific antigen that may represent various immune challenges (i.e., viruses, bacteria, and toxins) and subsequently trigger appropriate immune responses, B cell requires functional rearrangement and repertoire diversification of the B-cell receptor (BCR), an immunoglobulin molecule consisting of two IGHs paired with two IGKs or IGLs. Analogous to the BCR, the T-cell receptor (TCR) dictates the role and functionality of T cell-mediated immune responses. Overall, receptor diversity is estimated to range from 10^14^ to 10^18^ and 10^11^ to 10^15^ for the BCR (Elhanati et al., 2015; Yaari and Kleinstein, 2015) and the TCR, respectively (Davis and Bjorkman, 1988; Jenkins et al., 2010). The uniqueness and specificity of both the BCR and TCR have enabled their use as individual identifiers of B and T cells (van Dongen et al., 2003). This is particularly true for the identification of monoclonality, a feature of cancer where malignant cells are derived from a common progenitor (Nowell, 1976; Tanooka, 1988).

As reported by van Dongen et al. (2003), PCR-based clonality analysis has a remarkable detection limit of 1%, with some rearrangement detected at <1%. Indeed, molecular testing based on receptor clonality has greatly advanced the field of VRL diagnosis. For example, Coupland et al. (2005b) demonstrated the existence of an “oculocerebral” lymphoma and established the underlying association between VRL and PCNSL based on identical IGH clonality in cells derived from the eye and brain. In a landmark paper by Wang et al. (2011), the researchers demonstrated that clonality testing is a reliable biomarker for both B- and T-cell VRL; when combined with microdissection, they yielded VRL diagnoses of nearly 100% sensitivity and specificity, rendering the approach superior to cytology and cytokine analysis.

Nonetheless, discretion is still needed as molecular testing could also give rise to false negatives or false positives. False negatives, while uncommon, can occur when clonal DNA of a malignant cell is not detected due to insufficient primer coverage, improper primer binding, and/or poor DNA quantity and quality (White et al., 1999; Baehring et al., 2005; van Krieken et al., 2007). False positives are more common and are attributable to the low cellularity of vitreous and cerebrospinal fluid biopsies (Zhou et al., 1999). In such cases, cellular paucity prevents the true elucidation of receptor diversity indicative of polyclonality and instead misrepresents scantiness as pseudo-monoclonality. Indeed, false positives have been demonstrated experimentally by Elenitoba-Johnson et al. (2000), where IGHs of an identical size were only identified as two different clones upon sequencing. Additionally, a false positive diagnosis could arise in immune-privileged sites, such as the eye, where compartmentalized B-cell expansion occurs in response to infections (Klaren and Peek, 2001; Peek et al., 2002) or immunologic disorders (Colombo et al., 2000; Bonzheim et al., 2015). While many others have suggested that PCR amplicons reproduced in two or more independent IGH clonality assays could be reliably interpreted as derivatives from monoclonal B cells, such an approach is counterintuitive. This is because B-cell clonality—the pairing of an immunoglobulin heavy chain (IGH, chromosome 14) and light chain kappa (IGK, chromosome 2) or lambda (IGL, chromosome 22) on each individual B cell—cannot be detected using conventional clonality assays, as they rely on a mixed DNA source from bulk samples, not individual cells. Going forward, we anticipate a need to characterize immunoglobulin pairings (IGH:IGK or IGH:IGL) at single B-cell resolution for complete clonality analysis in vitreous biopsies.

#### B-Cell Lymphoma 2 (BCL2)

A BCL2/JH t(14;18) translocation is an aberrant chromosomal translocation event where a segment of chromosome 18 encoding the antiapoptotic gene BCL2 is juxtaposed next to the IGH locus on chromosome 14. This translocation leads to a BCL2 gain of function and an increase in the level of BCL2 protein expression (Scarfo and Ghia, 2013; Zhang et al., 2018), enabling cells to overcome apoptosis (Vaux et al., 1988). The BCL2/JH t(14;18) translocation is a cytogenetic hallmark of follicular lymphoma, being found in 57–90% of affected patients (Yunis et al., 1987). This translocation is also found in ~30% of DLBCL cases (Weiss et al., 1987; Sakruti et al., 2013; Akkaya et al., 2016), albeit more commonly in GCB DLBCL than ABC DLBCL (Barrans et al., 2002; Iqbal et al., 2004; Chen et al., 2010). While fluorescence *in situ* hybridization is frequently used to detect the BCL2 translocation in follicular lymphoma, PCR is generally used to detect this translocation in VRL.

VRL with an underlying BCL2 translocation was first reported by White et al. (1999) in 1993 and was later reported to be found in 57% of patients with VRL (Wallace et al., 2006). Given that most VRL cases are of the ABC DLBCL subtype (Araujo and Coupland, 2017; Karakawa et al., 2018; Chen et al., 2019; Fan et al., 2019) and that BCL2 is more commonly found in GCB DLBCL (Barrans et al., 2002; Iqbal et al., 2004; Chen et al., 2010), the high occurrence of the BCL2 translocation in patients with VRL as reported by Wallace et al. (2006) is intriguing (Fend et al., 2016; Kalogeropoulos et al., 2019). In a cohort of 106 patients with DLBCL, both overall survival and progression-free survival were significantly poorer in 27 patients who had the BCL2 translocation (Zhang et al., 2011). This finding is consistent with a report by Kaminski et al. (2005), who found that patients negative for the BCL2 translocation had better clinical outcomes and a more durable, complete remission than those with the translocation. Despite the potential utility of BCL2 translocation analysis to help stratify patients with DLBCL for appropriate disease management (Hwang et al., 2001; Muris et al., 2006; Kendrick et al., 2014; Kawamoto et al., 2016), BCL2 translocation status is not considered to be a diagnostic biomarker of VRL.

#### Myeloid Differentiation Primary Response 88 (MYD88)

MYD88 is an adaptor protein that activates NF-κB (nuclear factor κ-light-chain-enhancer of activated B cells) expression *via* Toll/IL-1 signaling (Deguine and Barton, 2014). Constitutive NF-κB activation is central to ABC DLBCL pathogenesis and is found in 69–82% of PCNSL cases (Kraan et al., 2013; Braggio et al., 2015; Nakamura et al., 2016) and VRL cases (Bonzheim et al., 2015; Raja et al., 2016; Hiemcke-Jiwa et al., 2018; Miserocchi et al., 2018; Yonese et al., 2018), where a single nucleotide mutation leads to a leucine (L) to proline (P) amino acid substitution at position 265 of the MYD88 protein (MYD88^L265P^). Despite being one of the most recently identified disease biomarkers for VRL, MYD88^L265P^ mutation testing can be achieved *via* a myriad of molecular applications. Originally, the MYD88^L265P^ mutation was detected by PCR coupled with Sanger sequencing, but this method was found to have a poor detection sensitivity of just 25%. To improve the sensitivity of MYD88^L265P^ mutation detection, Wang et al. (2013) used high-resolution melt analysis (HRMA)—a fast and cost-effective post-PCR screening approach based on a distinctive melting temperature profile of different nucleotide sequences (Li et al., 2011; Wang et al., 2013). However, HRMA was soon replaced by allele-specific PCR (AS-PCR) due to its superior detection limit (0.1–1.25%) and compatibility for use with highly degraded DNA isolated from FFPE samples (Xu et al., 2013; Staiger et al., 2015). Notably, the combination of AS-PCR with melt-curve analyses resulted in the correction of a misdiagnosis in a patient with HIV who was initially diagnosed with VRL, instead of viral retinitis, based on B-cell clonality (Bonzheim et al., 2015). Now, even newer molecular techniques, such as ddPCR—where PCR is subdivided into nanoliter partitions where confined target amplification can be enumerated independently—are used to detect the MYD88^L265P^ mutation. In VRL, ddPCR has shown 100% specificity for MYD88^L265P^ detection in the vitreous fluid and aqueous humor (Hiemcke-Jiwa et al., 2018). In PCNSL, the method has also successfully detected the MYD88^L265P^ mutation in cell-free DNA obtained from the serum of patients with PCNSL (Hattori et al., 2018).

**Figure 1:**
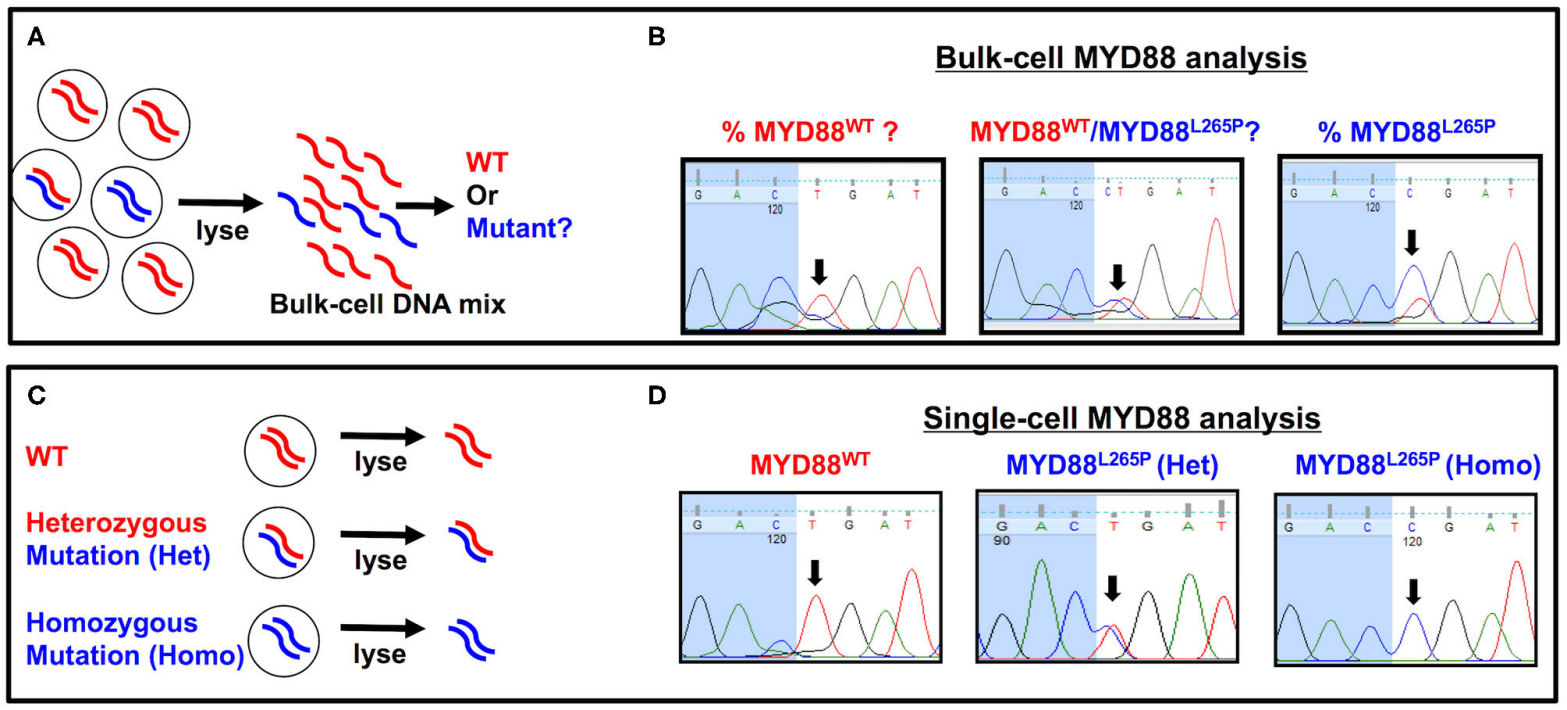
Comparison of bulk cell-vs. single cell-based MYD88 analysis. **(A)** A heterogeneous cellular sample with MYD88^WT^ (red) or MYD88^L265P^ mutants (blue) was lysed for bulk-cell myeloid differentiation primary response 88 (MYD88) analysis. **(B)** An electropherogram showing sequencing peaks for MYD88^WT^ and MYD88^L265P^. The black arrows throughout indicate the location of a T→C point mutation corresponding to MYD88^WT^ (C***T***G) or MYD88^L265P^ (C***C***G), respectively. **(C)** Individual cells with MYD88^WT^ (red/red), heterozygous (red/blue), or homozygous MYD88^L265P^ mutant (blue/blue) were isolated and lysed for single cell-based MYD88 analysis. **(D)** An electropherogram showing clean and well-resolved sequencing peaks for MYD88^WT^, heterozygous (red/blue), or homozygous MYD88^L265P^ alleles from the single cells. The black arrows throughout indicate the location of a T→C point mutation corresponding to MYD88^WT^ (C***T***G) or MYD88^L265P^ (C***C***G), respectively. WT, wild-type MYD88^WT^; mutant, MYD88^L265P^.

In summary, the ultrasensitive capability of ddPCR not only provides a promising tool for VRL diagnosis but also highlights the possibility of non-invasive diagnostics (Hattori et al., 2018; Hiemcke-Jiwa et al., 2018; Shi et al., 2019). While the progressive improvements to PCR-based approaches seem to have diminished the utility of Sanger sequencing in VRL diagnosis, we recently demonstrated the relevance of Sanger sequencing when paired with single-cell technology. Specifically, we found that this pairing can help to characterize the genotype of individual B cells as being MYD88 wild-type (MYD88^WT^) or MYD88^L265P^ heterozygous or homozygous mutants (Tan et al., 2019). Single cell-based MYD88 analysis has several advantages over bulk cell-based approaches. First, interpreting the results of bulk cell-based MYD88 analysis may not always be straightforward. The sequencing peaks of MYD88^WT^ and MYD88^L265P^ may appear together (evenly or unevenly) in bulk-cell DNA mixtures with heterogeneous MYD88 profiles (Figures 1A,B). Although sequencing peaks of MYD88^WT^ and MYD88^L265P^ with uneven amplitudes may indicate the presence of both alleles (with one more dominant than the other), the proportion of MYD88^WT^ to MYD88^L265P^ is difficult to determine precisely from bulk-cell analysis (Figure 1B). On the other hand, sequencing peaks of the same amplitude may suggest an equal ratio of MYD88^WT^ to MYD88^L265P^ (Figure 1B, center panel) but does not reflect the MYD88 zygosities (MYD88^WT^ or MYD88^L265P^ heterozygous or homozygous alleles) in individual cells. In contrast, single-cell MYD88 analysis is able to reveal clean and well-resolved sequencing peaks corresponding to MYD88^WT^, heterozygous, or homozygous MYD88^L265P^ alleles in individual cells (Figures 1C,D), providing a useful genetic tool to aid VRL diagnosis (Tan et al., 2019). To the best of our knowledge, no other technique currently compares with MYD88^L265P^ zygosity and mutation analysis for VRL diagnosis in terms of sensitivity or specificity. Nonetheless, a study by Bonzheim et al. (2015) demonstrated that the implementation of MYD88^L265P^ analysis confirmed a diagnosis of VRL in six cases initially diagnosed as either reactive or suspicious for lymphoma, based on cytology, immunohistochemistry (IHC), and clonality analysis. This increased the diagnostic sensitivity from 62 to 90.5% and the negative predictive value from 85.5 to 96%, while also maintaining diagnostic specificity (Bonzheim et al., 2015). While reports on the use of the MYD88^L265P^ mutation for VRL diagnosis are largely supportive, it should be noted that 20–30% of patients with VRL are negative for this mutation, cautioning against its use as a stand-alone diagnostic tool (Yonese et al., 2018). Patients must be managed using multifarious diagnostic strategies, ranging from cytological IHC to B-cell clonality and cytokine analysis, to confirm VRL negativity.

The presence of oncogenic MYD88 mutations have produced further clinical insights, providing potential drug targets that could inhibit MYD88-driven DLBCL pathogenesis. For example, ibrutinib, a small-molecule drug that targets Bruton's tyrosine kinase (BTK), can be considered for the treatment of patients with VRL who have an underlying MYD88^L265P^ mutation, which is known to trigger lymphomagenesis *via* BTK activation. Although there is currently no clear correlation between MYD88 mutational profiles and ibrutinib response (Visco et al., 2020), several studies have shown clinical responses to ibrutinib treatment in some DLBCL patients with an MYD88^L265P^ mutation (Deng et al., 2017; Soussain et al., 2019). MYD88^L265P^ mutations frequently co-occur in DLBCL patients who also harbor a mutation of CD79B, a subunit that is important for transducing BCR signaling. Interestingly, DLBCL patients with concomitant MYD88 and CD79B mutations showed impressive clinical responses (>80%) to ibrutinib (Wilson et al., 2015; Grommes et al., 2017). The hypersensitivity of MYD88/CD79B double-mutant DLBCLs to ibrutinib treatment is probably due to the co-inhibition of BTK and CD79B-dependent BCR signaling. Taken together, oncogenic mutations of MYD88 and CD79B in DLBCL have major implications for clinical practice (Visco et al., 2020) and may provide useful genetic tools for personalized targeted therapies.

### Single-Cell Analyses

Cells were traditionally classified based on their shape, function, location, and interaction with other cell types; little attention was paid to cellular heterogeneity at the molecular level. Additionally, analyses were conducted on bulk samples, with the results based on weighted averages. However, this approach would mask the influence of underrepresented minority cells that might constitute a critical pathogenic cell population. With the advent of single-cell analyses (SCA) in 2013, molecular insights into seemingly identical cells were made possible (Elizabeth, 2012). Today, SCA is widely used in fields such as microbiology, immunology, and oncology. The importance of SCA is further underscored by the Human Cell Atlas initiative (London, 2016)—a global collaborative effort to characterize cells in health and in disease using SCA as the driving force. The aim of this initiative is to define and map cell types based on their molecular signatures. Similar projects, such as the Human Retina Atlas (Lukowski et al., 2019; Menon et al., 2019) and The Cancer Genome Atlas (Cancer Genome Atlas Research et al., 2013; Kandoth et al., 2013), demonstrate the level of enthusiasm across various fields for SCA. In ophthalmology, combining SCA with RNA sequencing [otherwise known as single-cell RNA sequencing (scRNA-Seq)] has helped us to study retinal development (Welby et al., 2017; Hu et al., 2019). So far, single B- and T-cell repertoire sequencing of the cerebrospinal fluid (CSF) has been carried out for diseases such as glioma and multiple sclerosis but not for PCNSL or VRL (Lanz et al., 2019). We anticipate that such analyses will give detailed unbiased overviews of clonal evolution and provide critical insights into the disease mechanisms underlying PCNSL and VRL.

Indeed, an area that we feel has thus far been neglected is the application of SCA for VRL diagnosis. Although single-cell technologies have been available for a long time, studies utilizing SCA are few and far between. To date, most molecular testing for VRL diagnosis has been performed on bulk samples, with no targeted selection of B cells or malignant cells. The pioneering use of SCA was conducted by Chan et al. (1998) and Shen et al. (1998) in 1998. These researchers micro-dissected and isolated neoplastic cells in combination with PCR and successfully demonstrated monoclonal IGH rearrangement in frozen and FFPE ocular specimens from patients with PCNSL. Subsequently, in 2011, the same researchers used the same approach and reported almost 100% sensitivity and specificity, obtaining a higher diagnostic value than either cytology or cytokine analysis (Tuaillon and Chan, 2001; Wang et al., 2011).

Our group has also leveraged SCA and elucidated the heterogeneity of MYD88 zygosity in single B cells isolated from patients with VRL (Tan et al., 2019). While it remains to be clarified whether the gene dosage of MYD88 expression (MYD88^L265P^ heterozygous vs. MYD88^L265P^ homozygous) and NF-κB activation have any effect on disease presentation and progression, our approach has nonetheless provided a means to determine the disease burden. It is also conceivable that SCA could supplement cytological examinations to improve the accuracy of current VRL diagnostics, especially in atypical cases.

Going forward, molecular analyses could help solve some of the most pertinent questions in PCNSL and VRL biology. The etiologies of PCNSL and VRL are elusive, with both infectious and hematogenous theories having been proposed to explain the origin of these lymphomatous cells (Faia and Chan, 2009). While it seems challenging if not impossible to prove these theories, the application of SCA would help to dissect the association of PCNSL and VRL in patients with concurrent involvements, provided that biopsies from both sites are available. In such instances, single-cell DNA sequencing could help to elucidate the relationships between cells from the CNS and the eye. This concept is based on the understanding that tumorigenesis (and metastasis) is the result of a steady accumulation of mutations, which by virtue could serve as a molecular clock for tracking cancer evolution. Coupling SCA with DNA sequencing and bioinformatics analyses could help deconstruct and then reconstruct these molecular events, thereby establishing the origins and relatedness of cells from the two anatomical sites. Such an approach has been used for other cancers, so there is good reason to think that this strategy could also be applied to PCNSL and VRL (Ley et al., 2008; Gawad et al., 2014).

Of note is the capability of SCA to uncover intratumor heterogeneity; this technique is helping change the perspective that cancer is a homogeneous manifestation. For example, SCA has helped to identify drug-resistant cellular subgroups within tumors, providing an explanation for disease relapse and treatment failure in many patients with cancer (Laughney et al., 2014). Additionally, SCA has enabled survival predictions to be made in the context of breast and lung cancers (Shi et al., 2018). Such applications could also be relevant to PCNSL and VRL.

Despite these great advantages of SCA, there are some notable limitations to consider. For example, SCA confers a long experimental time, high costs, and requires a bioinformatics analytical pipeline to handle the large quantity of data produced. These are all notable factors against adopting SCA on a routine basis. As technology continues to advance, however, single-cell sequencing platforms are becoming increasingly automated and multidimensional, with higher throughputs and better sensitivities achieved at lower costs. At the same time, advances in the field of artificial intelligence and machine learning algorithms could help mitigate the gap between biological research and data science. All such developments could potentiate the use of SCA on a broader scale and thereby increase the relevance of SCA for VRL diagnostics in clinical settings.

## Discussion

Making a prompt and accurate VRL diagnosis is essential, as 65–90% of affected patients eventually progress to PCNSL, an aggressive lymphoma that confers a high mortality rate (Chan et al., 2011; Ponzoni, 2015). At present, cytology is the gold standard for VRL diagnosis, and other techniques, including cytokine analyses, flow cytometry, and molecular testing, remain adjunctive. Limitations of these adjunctive techniques include that the criteria (e.g., IL-10:IL-6 >1, monotypic expression, and clonal rearrangement) for a diagnosis are not exclusive to VRL, and they are by no means definitive. Furthermore, the VRL indication for each method varies for different sample types, and the diagnostic performance depends on the sample quality as well as the availability of specialized laboratory equipment (Supplementary Table 1).

Despite potential limitations, molecular analysis still holds immense potential for VRL diagnostics. Indeed, the advent of molecular biology has greatly expanded our understanding of PCNSL and VRL. In particular, the application of molecular gene expression profiling enabled us to identify clinical subtypes of DLBCL and to gain insights as to how these molecular entities differ in terms of disease progression, prognosis, and treatment responses (Alizadeh et al., 2000; Nowakowski and Czuczman, 2015). More recently, the emergence of the MYD88^L265P^ mutation has gained much attention for VRL diagnosis, as it can greatly enhance diagnostic accuracy and confirm VRL in cases initially misdiagnosed by cytology (Bonzheim et al., 2015). The successful detection of the MYD88^L265P^ mutation in cell-free DNA circulating in the blood (Hattori et al., 2018) also highlights the possibility of developing non-invasive diagnostics in the near future, which would revolutionize CNSL and VRL diagnosis.

Although PVRL is classified as a subset of PCNSL, it can manifest with or without CNS involvement. In this regard, questions remain as to whether PVRL and PCNSL are synonymous or subtypes that could be further differentiated by their molecular signatures. While the ABC DLBCL subtype is widely recognized in both PCNSL and VRL (Camilleri-Broet et al., 2006; Bhagavathi et al., 2008; Cheng et al., 2008; Montesinos-Rongen et al., 2009; Hattab et al., 2010; Li et al., 2017; Belhouachi et al., 2020), many have independently reported the detection of GCB DLBCL in PCNSL and VRL (Bhagavathi and Wilson, 2008; Montesinos-Rongen et al., 2008; Wang, 2017; Arai et al., 2020), adding discordance to our current understanding. Admittedly, the classification of DLBCL based on gene expression profiling (Alizadeh et al., 2000) and IHC (Hans's algorithm) (Hans et al., 2004) was developed almost two decades ago; more importantly, they were not formulated to classify immune-privileged lymphomas such as PCNSL or VRL. For this reason, newer classifications based on data from larger study cohorts and incorporating more defined genetic markers have duly enhanced the classification of PCNSL and VRL (Chapuy et al., 2018; Schmitz et al., 2018; Lacy et al., 2020). In these new classifications, the MYD88^L265P^ mutation was used to characterize a distinct molecular subtype (Schmitz et al., 2018; Lacy et al., 2020); this mutation is consistently found in PCNSL and testicular lymphoma, substantiating its reliability as a diagnostic biomarker for immune-privileged lymphomas (Kraan et al., 2013; Chapuy et al., 2016; Nayyar et al., 2019).

We have also seen the application of next-generation sequencing to analyze remnant vitreous biopsies following standard cytological examination and flow cytometry analyses. With as little as 5.8 ng of DNA, Cani et al. (2017) simultaneously detected the MYD88^L265P^ mutation and loss of tumor suppressor CDKN2A expression.

Single-cell technologies have been widely applied across different biomedical fields over the past decade and have great potential for guiding precision diagnosis of VRL. The ability to distinguish and isolate rare target lymphoma cells from paucicellular vitreous biopsies poses unique challenges that impede the accuracy of VRL diagnosis. Most of the mainstream single-cell technologies (e.g., FACS, 10× Genomics, Drop-Seq, etc.) require samples with high cellularity for phenotyping and molecular characterization. However, the number of cells typically available from the vitreous biopsies used for VRL diagnosis is limited and heterogeneous. Recently, we provided a proof-of-concept that imaging-based digital sorting DEPArray™ NxT technology can isolate rare B cells in vitreous biopsies for single-cell MYD88 sequencing to aid VRL diagnosis (Tan et al., 2019). Going forward, high-resolution single-cell studies are warranted to examine and validate the association between MYD88^L265P^ mutation status and VRL and CNSL pathogenesis in a larger cohort, particularly in paired samples from patients with VRL (vitreous or choroid) and from elsewhere in the CNS (brain, spinal cord, and cerebrospinal fluid).

In summary, the current standing of bulk-cell molecular testing serves as no more than an adjunctive to cytological examination. Single-cell technologies are increasingly being adopted for precision diagnostic applications. With the wealth of information that stands to be extracted, it is foreseeable that single cell-based analysis has the potential to provide clinically relevant solutions for precision diagnostics for VRL in the near future.

## Author Contributions

WJT and TSL wrote the manuscript, and all authors reviewed and approved the submission of the manuscript.

## Conflict of Interest

WJT and TSL were employees and PR-C a consultant for A. Menarini Biomarkers Singapore Pte Ltd. ASYC received research funding from A. Menarini Biomarkers Singapore Pte Ltd. The remaining author declares that the research was conducted in the absence of any commercial or financial relationships that could be construed as a potential conflict of interest.
