# Supplementary material for: Cytologic and Molecular Diagnostics for Vitreoretinal Lymphoma: Current Approaches and Emerging Single-Cell Analyses
Source: Front Mol Biosci. 2021 Jan 11;7:611017. doi: 10.3389/fmolb.2020.611017 (PMC7832476; doi:10.3389/fmolb.2020.611017)
Supplement: Supplementary file 1 [file Table_1.DOCX]

| **Supplementary Table 1. Summary of biomarkers, types, indication, diagnostics performance in vitreoretinal lymphoma** | | | | | | |
| --- | --- | --- | --- | --- | --- | --- |
| **Biomarkers** | **Types** | **Method** | **VRL Indication** | **^#^Diagnostic Performance** | **Remarks** | **Reference** |
| CD20, PAX5, CD79a, Ki-67 | B-cell markers | Cytology +  IHC | Large, atypical lymphoid cells expressing CD20, PAX5, CD79a with proliferating activity (Ki-67^+^ cells > 80%) | Sens: 0.45-0.60 (Cytology alone);  Sensitivity of cytology is enhanced by IHC staining | Requires specialized examination from pathologists;  Depends on quality of vitreous biopsies; | (Coupland et al., 2003; Fend et al., 2016) |
| CD3, CD19, CD20, CD45,  Ig$\kappa$, Ig$\lambda$ | Surface marker | Flow Cytometry | CD45^+^, CD3^-^, CD20^+^ CD20^+^ B-cells with disequilibrium Ig$\kappa$ versus Ig$\lambda$ expression | Sens=0.82; Spec=1.00; | Requirement of large amount of cells (relative to cytology)  Reported losses of surface immunoglobulin light chain in VRL cases;  Presence of immunoreactive B and T-cells; | (Davis et al., 1997; Horna et al., 2011; Davis et al., 2012; Davis, 2013; Missotten et al., 2013) |
| IL-10 | Soluble | ELISA | >400pg/mL(vitreous)  >65pg/mL (vitreous) | Sens=0.99, Spec=0.80;  Sens=0.89, Spec=0.93 | Varying cut-off value of IL-10 concentration in different studies; | (Cassoux et al., 2007)  (Pochat-Cotilloux et al., 2018) |
|  | Soluble | ELISA | >50pg/mL (aqueous)  >30pg/mL (aqueous)  >41.5pg/mL(aqueous) | Sens=0.93, Spec=1.00;  Sens=0.78, Spec=0.97  Sens=0.88, Spec=1.00 | High sensitivity and specificity value provides good indication;  Sources of IL-10 and IL-6-secreating cells are unknown; | (Cassoux et al., 2007)  (Pochat-Cotilloux et al., 2018)  (Kuiper et al., 2017) |
| IL-10:IL-6 | Soluble | ELISA | IL-10:IL-6 ratio > 1 | Sens=0.75, Spec=0.74  Sens=0.81, Spec=1.00  Sens=0.88, Spec=0.85  Sens=0.93, Spec=1.00 | Cytokine levels could be affected by corticosteroid and immunosuppressant treatments  IL-10: IL-6 < 1 reading does not exclude VRL; 60% of non-neoplastic uveitis had IL-10: IL-6 ratio >1 | (Wolf et al., 2003)  (Sugita et al., 2009)  (Wang et al., 2011)  (Pochat-Cotilloux et al., 2018)  (Akpek et al., 1999) |
|  |  | ELISA | *ISOLD >4.6 | Sens=0.93; Spec=0.95 | Useful adjunctive tool to support VRL diagnosis | (Costopoulos et al., 2016) |
| IFN-$\gamma$, CCL2,  CCL3, CCL4,  CXC12, CXC13,  VEGF-A, IL-2R$\alpha$  IL-6R, TNF-R1, TNFR2,VEGFR1,  VEGFR2, | Soluble cytokines, growth factors, receptors | Multiplexed ELISA | Changes level in VRL | N.A. | Additional studies needed to establish diagnostic use | (Usui et al., 2012; Fisson et al., 2013; Caraballo et al., 2014; Takeda et al., 2015) |
| miR-155 | microRNA | miRNA microarray | lower in VRL;  higher in uveitis | N.A. | No consensus of differential expressed microRNA. | (Tuo et al., 2014) |
| miR-19b, miR-21,  miR-92 | microRNA | TaqMan  miRNA assay | higher in VRL;  lower in uveitis | N.A. | No consensus of differential expressed microRNA. | (Kakkassery et al., 2017) |
| IGH | Bulk cell | PCR | Single PCR amplicon | Sens=0.95; Spec=1.00  Sens=0.96; Spec=1.00  Sens=1.00; Spec=0.99 | False negative due to poor DNA quantity and quality, or insufficient primers coverage and improper binding;  False positive due to presence of pseudoclonal PCR bands; | (Lobo et al., 2007) (Sugita et al., 2009)  (Wang et al., 2011) |
| BCL2/JH t(14;18) | Bulk cell | Chromosomal translocation detected by PCR | Presence of BCL2/JH t(14;18) | Sens=0.57  Sens=0.25 | Low sensitivity of detection  Detectable in healthy subjects | (Wallace et al., 2006; Lobo et al., 2007)  (Limpens et al., 1995) |
| MYD88 | Bulk cell | Allele-specific PCR +/- melt curve analysis | Presence MYD88^L265P^ mutation | Sens: 0.62  (cyto only)  Sens: 90.5;  (cyto+MYD88) | Supplementation of MYD88^L265P^ analysis increases diagnostic sensitivity from 62% to 90.5%, maintaining diagnostic specificity  Binary detection of MYD88^L265P^.  Unable to characterize percentage of wild type vs MYD88^L265P^ mutant cells | (Bonzheim et al., 2015; Pulido et al., 2016)  (Li et al., 2011; Wang et al., 2013; Xu et al., 2013; Bonzheim et al., 2015; Staiger et al., 2015) |
| MYD88 | Cell-free DNA | Droplet digital PCR (ddPCR) | Presence of MYD88^L265P^ mutation | Sens: 0.67 (aqueous); 0.75 (vitreous)  Spec: 1.00 | Detectable in small volume of minimally invasive aqueous sample; | (Hiemcke-Jiwa et al., 2018)  (Hattori et al., 2018) |
| MYD88 | Single cell | DEPArray +PCR | > 5% B-cells expressing homozygous MYD88^L265P^ | N.A. | Able to sort and characterize MYD88 zygosity of single B cells from paucicellular vitreous biopsies;  Requires specialized equipment setup | (Tan et al., 2019) |
| PTPRK, CDKN2A | Bulk cell | SNP microarray | Copy number loss | N.A. | Additional studies needed to establish diagnostic use | (Wang et al., 2014) |
| PTEN, CDKN2A | Bulk cell | NGS | Copy number loss | N.A. | Additional studies needed to establish diagnostic use | (Cani et al., 2017) |

# Sens: Sensitivity; Spec: Specificity; *ISOLD: Interleukin Score for intra-Ocular Lymphomas Diagnosis, a mathematical formula with IL-10 and IL-6 concentrations; VRL: vitreoretinal lymphoma; IHC: immunohistochemistry; ELISA: enzyme-linked immunosorbent

**References**

Akpek, E.K., Maca, S.M., Christen, W.G., and Foster, C.S. (1999). Elevated vitreous interleukin-10 level is not diagnostic of intraocular-central nervous system lymphoma. *Ophthalmology* 106(12)**,** 2291-2295. doi: 10.1016/s0161-6420(99)90528-6.

Bonzheim, I., Giese, S., Deuter, C., Susskind, D., Zierhut, M., Waizel, M., et al. (2015). High frequency of MYD88 mutations in vitreoretinal B-cell lymphoma: a valuable tool to improve diagnostic yield of vitreous aspirates. *Blood* 126(1)**,** 76-79. doi: 10.1182/blood-2015-01-620518.

Cani, A.K., Hovelson, D.H., Demirci, H., Johnson, M.W., Tomlins, S.A., and Rao, R.C. (2017). Next generation sequencing of vitreoretinal lymphomas from small-volume intraocular liquid biopsies: new routes to targeted therapies. *Oncotarget* 8(5)**,** 7989-7998. doi: 10.18632/oncotarget.14008.

Caraballo, J.N., Snyder, M.R., Johnston, P.B., BP, O.N., Raja, H., Balsanek, J.G., et al. (2014). Vitreoretinal lymphoma versus uveitis: cytokine profile and correlations. *Ocul Immunol Inflamm* 22(1)**,** 34-41. doi: 10.3109/09273948.2012.752507.

Cassoux, N., Giron, A., Bodaghi, B., Tran, T.H., Baudet, S., Davy, F., et al. (2007). IL-10 measurement in aqueous humor for screening patients with suspicion of primary intraocular lymphoma. *Invest Ophthalmol Vis Sci* 48(7)**,** 3253-3259. doi: 10.1167/iovs.06-0031.

Costopoulos, M., Touitou, V., Golmard, J.L., Darugar, A., Fisson, S., Bonnemye, P., et al. (2016). ISOLD: A New Highly Sensitive Interleukin Score for Intraocular Lymphoma Diagnosis. *Ophthalmology* 123(7)**,** 1626-1628. doi: 10.1016/j.ophtha.2016.01.037.

Coupland, S.E., Bechrakis, N.E., Anastassiou, G., Foerster, A.M., Heiligenhaus, A., Pleyer, U., et al. (2003). Evaluation of vitrectomy specimens and chorioretinal biopsies in the diagnosis of primary intraocular lymphoma in patients with Masquerade syndrome. *Graefes Arch Clin Exp Ophthalmol* 241(10)**,** 860-870. doi: 10.1007/s00417-003-0749-y.

Davis, J.L. (2013). Intraocular lymphoma: a clinical perspective. *Eye* 27(2)**,** 153-162. doi: 10.1038/eye.2012.250.

Davis, J.L., Ruiz, P., Jr., Shah, M., and Mandelcorn, E.D. (2012). Evaluation of the reactive T-cell infiltrate in uveitis and intraocular lymphoma with flow cytometry of vitreous fluid (an American Ophthalmological Society thesis). *Trans Am Ophthalmol Soc* 110**,** 117-129.

Davis, J.L., Viciana, A.L., and Ruiz, P. (1997). Diagnosis of intraocular lymphoma by flow cytometry. *Am J Ophthalmol* 124(3)**,** 362-372. doi: 10.1016/s0002-9394(14)70828-1.

Fend, F., Ferreri, A.J., and Coupland, S.E. (2016). How we diagnose and treat vitreoretinal lymphoma. *Br J Haematol* 173(5)**,** 680-692. doi: 10.1111/bjh.14025.

Fisson, S., Ouakrim, H., Touitou, V., Baudet, S., Ben Abdelwahed, R., Donnou, S., et al. (2013). Cytokine profile in human eyes: contribution of a new cytokine combination for differential diagnosis between intraocular lymphoma or uveitis. *PLoS One* 8(2)**,** e52385. doi: 10.1371/journal.pone.0052385.

Hattori, K., Sakata-Yanagimoto, M., Suehara, Y., Yokoyama, Y., Kato, T., Kurita, N., et al. (2018). Clinical significance of disease-specific MYD88 mutations in circulating DNA in primary central nervous system lymphoma. *Cancer Sci* 109(1)**,** 225-230. doi: 10.1111/cas.13450.

Hiemcke-Jiwa, L.S., Ten Dam-van Loon, N.H., Leguit, R.J., Nierkens, S., Ossewaarde-van Norel, J., de Boer, J.H., et al. (2018). Potential Diagnosis of Vitreoretinal Lymphoma by Detection of MYD88 Mutation in Aqueous Humor With Ultrasensitive Droplet Digital Polymerase Chain Reaction. *JAMA Ophthalmol* 136(10)**,** 1098-1104. doi: 10.1001/jamaophthalmol.2018.2887.

Horna, P., Olteanu, H., Kroft, S.H., and Harrington, A.M. (2011). Flow cytometric analysis of surface light chain expression patterns in B-cell lymphomas using monoclonal and polyclonal antibodies. *Am J Clin Pathol* 136(6)**,** 954-959. doi: 10.1309/AJCP3C2QZZBPTMLB.

Kakkassery, V., Schroers, R., Coupland, S.E., Wunderlich, M.I., Schargus, M., Heinz, C., et al. (2017). Vitreous microRNA levels as diagnostic biomarkers for vitreoretinal lymphoma. *Blood* 129(23)**,** 3130-3133. doi: 10.1182/blood-2017-01-765180.

Kuiper, J.J., Beretta, L., Nierkens, S., van Leeuwen, R., Ten Dam-van Loon, N.H., Ossewaarde-van Norel, J., et al. (2017). An Ocular Protein Triad Can Classify Four Complex Retinal Diseases. *Sci Rep* 7**,** 41595. doi: 10.1038/srep41595.

Li, B.-S., Wang, X.-Y., Ma, F.-L., Jiang, B., Song, X.-X., and Xu, A.-G. (2011). Is High Resolution Melting Analysis (HRMA) Accurate for Detection of Human Disease-Associated Mutations? A Meta Analysis. *PLOS ONE* 6(12)**,** e28078. doi: 10.1371/journal.pone.0028078.

Limpens, J., Stad, R., Vos, C., de Vlaam, C., de Jong, D., van Ommen, G.J., et al. (1995). Lymphoma-associated translocation t(14;18) in blood B cells of normal individuals. *Blood* 85(9)**,** 2528-2536.

Lobo, A., Okhravi, N., Adamson, P., Clark, B.J., and Lightman, S. (2007). Protocol for the use of polymerase chain reaction in the detection of intraocular large B-cell lymphoma in ocular samples. *J Mol Diagn* 9(1)**,** 113-121. doi: 10.2353/jmoldx.2007.050121.

Missotten, T., Tielemans, D., Bromberg, J.E., van Hagen, P.M., van Lochem, E.G., van Dongen, J.J., et al. (2013). Multicolor flowcytometric immunophenotyping is a valuable tool for detection of intraocular lymphoma. *Ophthalmology* 120(5)**,** 991-996. doi: 10.1016/j.ophtha.2012.11.007.

Pochat-Cotilloux, C., Bienvenu, J., Nguyen, A.M., Ohanessian, R., Ghesquieres, H., Seve, P., et al. (2018). Use of a Threshold of Interleukin-10 and Il-10/Il-6 Ratio in Ocular Samples for the Screening of Vitreoretinal Lymphoma. *Retina* 38(4)**,** 773-781. doi: 10.1097/IAE.0000000000001922.

Pulido, J.S., Raja, H., Vile, R.G., Salomao, D.R., and Viswanatha, D.S. (2016). Mighty MyD88 in Health and Disease. *Retina* 36(3)**,** 429-431. doi: 10.1097/IAE.0000000000000921.

Staiger, A.M., Ott, M.M., Parmentier, S., Rosenwald, A., Ott, G., Horn, H., et al. (2015). Allele-specific PCR is a powerful tool for the detection of the MYD88 L265P mutation in diffuse large B cell lymphoma and decalcified bone marrow samples. *Br J Haematol* 171(1)**,** 145-148. doi: 10.1111/bjh.13369.

Sugita, S., Takase, H., Sugamoto, Y., Arai, A., Miura, O., and Mochizuki, M. (2009). Diagnosis of intraocular lymphoma by polymerase chain reaction analysis and cytokine profiling of the vitreous fluid. *Jpn J Ophthalmol* 53(3)**,** 209-214. doi: 10.1007/s10384-009-0662-y.

Takeda, A., Yoshikawa, H., Fukuhara, T., Hikita, S., Hijioka, K., Otomo, T., et al. (2015). Distinct Profiles of Soluble Cytokine Receptors Between B-Cell Vitreoretinal Lymphoma and Uveitis. *Invest Ophthalmol Vis Sci* 56(12)**,** 7516-7523. doi: 10.1167/iovs.15-17465.

Tan, W.J., Wang, M.M., Ricciardi-Castagnoli, P., Tang, T., Chee, S.P., Lim, T.S., et al. (2019). Single-cell MYD88 sequencing of isolated B cells from vitreous biopsies aids vitreoretinal lymphoma diagnosis. *Blood* 134(8)**,** 709-712. doi: 10.1182/blood.2019000022.

Tuo, J., Shen, D., Yang, H.H., and Chan, C.C. (2014). Distinct microRNA-155 expression in the vitreous of patients with primary vitreoretinal lymphoma and uveitis. *Am J Ophthalmol* 157(3)**,** 728-734. doi: 10.1016/j.ajo.2013.12.014.

Usui, Y., Wakabayashi, Y., Okunuki, Y., Kimura, K., Tajima, K., Matsuda, R., et al. (2012). Immune mediators in vitreous fluids from patients with vitreoretinal B-cell lymphoma. *Invest Ophthalmol Vis Sci* 53(9)**,** 5395-5402. doi: 10.1167/iovs.11-8719.

Wallace, D.J., Shen, D., Reed, G.F., Miyanaga, M., Mochizuki, M., Sen, H.N., et al. (2006). Detection of the bcl-2 t(14;18) translocation and proto-oncogene expression in primary intraocular lymphoma. *Invest Ophthalmol Vis Sci* 47(7)**,** 2750-2756. doi: 10.1167/iovs.05-1312.

Wang, C.Z., Lin, J., Qian, J., Shao, R., Xue, D., Qian, W., et al. (2013). Development of high-resolution melting analysis for the detection of the MYD88 L265P mutation. *Clin Biochem* 46(4-5)**,** 385-387. doi: 10.1016/j.clinbiochem.2012.11.007.

Wang, L., Sato-Otsubo, A., Sugita, S., Takase, H., Mochizuki, M., Usui, Y., et al. (2014). High-resolution genomic copy number profiling of primary intraocular lymphoma by single nucleotide polymorphism microarrays. *Cancer Sci* 105(5)**,** 592-599. doi: 10.1111/cas.12388.

Wang, Y., Shen, D., Wang, V.M., Sen, H.N., and Chan, C.C. (2011). Molecular biomarkers for the diagnosis of primary vitreoretinal lymphoma. *Int J Mol Sci* 12(9)**,** 5684-5697. doi: 10.3390/ijms12095684.

Wolf, L.A., Reed, G.F., Buggage, R.R., Nussenblatt, R.B., and Chan, C.C. (2003). Vitreous cytokine levels. *Ophthalmology* 110(8)**,** 1671-1672. doi: 10.1016/S0161-6420(03)00811-X.

Xu, L., Hunter, Z.R., Yang, G., Zhou, Y., Cao, Y., Liu, X., et al. (2013). MYD88 L265P in Waldenstrom macroglobulinemia, immunoglobulin M monoclonal gammopathy, and other B-cell lymphoproliferative disorders using conventional and quantitative allele-specific polymerase chain reaction. *Blood* 121(11)**,** 2051-2058. doi: 10.1182/blood-2012-09-454355.
